# How succulent leaves of Aizoaceae avoid mesophyll conductance limitations of photosynthesis and survive drought

**DOI:** 10.1093/jxb/ert314

**Published:** 2013-10-14

**Authors:** Brad S. Ripley, Trevor Abraham, Cornelia Klak, Michael D. Cramer

**Affiliations:** ^1^Department of Botany, Rhodes University, 6140 Grahamstown, South Africa; ^2^Bolus Herbarium, Department of Biological Sciences, University of Cape Town, 7701 Rondebosch, South Africa; ^3^Department of Biological Sciences, University of Cape Town, 7701 Rondebosch, South Africa

**Keywords:** Aizoaceae, Crassulaceae, Crassulacean acid metabolism, drought avoidance, leaf succulence, mesophyll conductance

## Abstract

In several taxa, increasing leaf succulence has been associated with decreasing mesophyll conductance (*g*_M_) and an increasing dependence on Crassulacean acid metabolism (CAM). However, in succulent Aizoaceae, the photosynthetic tissues are adjacent to the leaf surfaces with an internal achlorophyllous hydrenchyma. It was hypothesized that this arrangement increases *g*_M_, obviating a strong dependence on CAM, while the hydrenchyma stores water and nutrients, both of which would only be sporadically available in highly episodic environments. These predictions were tested with species from the Aizoaceae with a 5-fold variation in leaf succulence. It was shown that *g*_M_ values, derived from the response of photosynthesis to intercellular CO_2_ concentration (*A*:*C*_i_), were independent of succulence, and that foliar photosynthate δ^13^C values were typical of C_3_, but not CAM photosynthesis. Under water stress, the degree of leaf succulence was positively correlated with an increasing ability to buffer photosynthetic capacity over several hours and to maintain light reaction integrity over several days. This was associated with decreased rates of water loss, rather than tolerance of lower leaf water contents. Additionally, the hydrenchyma contained ~26% of the leaf nitrogen content, possibly providing a nutrient reservoir. Thus the intermittent use of C_3_ photosynthesis interspersed with periods of no positive carbon assimilation is an alternative strategy to CAM for succulent taxa (Crassulaceae and Aizoaceae) which occur sympatrically in the Cape Floristic Region of South Africa.

## Introduction

Leaf succulence has evolved in diverse phylogenetic lineages worldwide (Landrum, 2002; Ogburn and Edwards, 2010) and is common in plants subject to frequent drought. Succulents form a major component of the flora of the Succulent Karoo within the Greater Cape Floristic Region (GCFR; *sensu* Born *et al*., 2007) and leaf succulence (as opposed to stem succulence) is found in some 30 lineages within the Aizoaceae, Asphodelaceae, and Crassulaceae (Linder *et al.*, 2010). This region contains some 1200 species of Aizoaceae (Goldblatt and Manning, 2000), one clade of which has undergone very rapid and recent diversification (Klak *et al.*, 2004). The driving force behind this rapid radiation is thought to be the innovation of several key morphological characters, including succulent, subcylindrical to trigonous shaped leaves, in response to an increasingly arid environment at the end of the Miocene (Klak *et al.*, 2004). However, the physiological role of leaf succulence in the tolerance of drought has not been investigated in the Aizoaceae.

Succulent leaves have high water contents, and this, combined with elastic cell walls and apoplastic polysaccharides, allows leaves to avoid low leaf water potentials even after extensive dehydration (Nobel, 1988; von Willert *et al.*, 1992; Martin, 1994; Pimienta-Barrios *et al.*, 2002). This serves to buffer transpirational water loss, prolonging stomatal opening and positive gas exchange, the magnitude of which correlates with the degree of leaf succulence (Nobel, 1976; Acevedo *et al.*, 1983; Martin and Adams, 1987; Gravatt and Martin, 1992; Martin, 1994). Subsequent to stomatal closure, succulence and the possession of relatively impervious leaf cuticles prevents cellular dehydration and allows succulent leaves to endure long periods of drought (Hoffman *et al.*, 2009).

In addition to increasing water storage, leaf succulence is frequently associated with the use of some form of photosynthetic Crassulacean acid metabolism (CAM), including obligate CAM, CAM-idling, CAM-cycling, and flexible CAM/C_3_ systems (Veste *et al.*, 2001; Herrera, 2009). Leaf succulence and CAM may be mechanistically linked, or may have evolved independently to optimize water use strategies in arid environments (Ogburn and Edwards, 2010). The proposed dependence of leaf succulence on CAM arises from two considerations. First, dependence on CAM is thought to require the additional storage capacity for the C_4_ acids provided for by enlarged succulent cells (Gibson, 1982; Nelson *et al.*, 2005) associated with high vacuolar volumes (up to 98% of cell volume; Lüttge, 2004). Secondly, as leaf succulence increases, the mesophyll conductance (*g*_M_) for the diffusion of CO_2_ through hydrated succulent leaf tissue to the photosynthetic tissue decreases. In most C_3_ and C_4_ plants CO_2_ transfer across the mesophyll is largely through gaseous pathways, with *g*_M_ being determined by the ratio of chloroplast surface to leaf area and cell wall thickness (Tomás *et al.*, 2013). In contrast, the mesophyll of fully hydrated succulent leaves at least partially lacks gas spaces, and CAM may function to overcome *g*_M_ limitations on photosynthesis (Maxwell *et al.*, 1997; Gillon *et al.*, 1998; Griffiths *et al.*, 2008). CAM plants can tolerate extremely low *g*_M_ values, partially due to the capacity of phosphoenolpyruvate carboxylase (PEPc) to acquire HCO_3_^–^ (Maxwell *et al.*, 1997) and because nocturnal carboxylation is limited by PEP derived from starch metabolism, and not by CO_2_ supply from the atmosphere (Nelson and Sage, 2008). Reduced *g*_M_ is advantageous to CAM plants, as it limits daytime CO_2_ efflux from photosynthetic tissues ensuring high intercellular CO_2_ concentrations during the re-fixation of CO_2_ into the C_3_ cycle (Nelson *et al.*, 2005, 2008).

The extent to which increasing succulence reduces *g*_M_ is likely to be dependent on leaf anatomy and how photosynthetic tissue is distributed within the leaf. In some species, increasing succulence is via additional layers of chloroplast-containing mesophyll cells, such that photosynthetic tissue is distributed throughout the leaf in what has been termed ‘all cell succulence’ (von Willert *et al.*, 1992). This anatomy is likely to result in a reciprocal relationship between succulence and *g*_M_, and may account for the tight relationship of succulence to CAM in groups such as the Crassulaceae, Orchidaceae, and some other families (Teeri *et al.*, 1981; Winter *et al.*, 1983; Kluge *et al.*, 1991; Nelson *et al.*, 2005; Silvera *et al.*, 2005; Nelson and Sage, 2008). In contrast, increasing succulence in taxa such as the Aizoaceae is associated with an inner cortex of water-storing cells, or epidermal and other peripheral cells that are achlorophyllous, and has been termed ‘storage succulence’ (Ihlenfeldt, 1989). The chlorenchyma in these species is often distributed around the periphery of the leaves and is distinct from the water-storing hydrenchyma (Ernshaw *et al.*, 1987). Many storage succulents do not show a strong dependence on CAM and assimilate the majority of their carbon diurnally through the C_3_ cycle (Rundel *et al.*, 1999; Winter and Holtum, 2002). These species may not benefit from reduced *g*_M_, which would limit their diurnal CO_2_ uptake. The anatomy of storage succulence suggests that they may acquire the benefit of stored water, without becoming *g*_M_ limited and may thus be less strongly dependent on CAM. This reasoning suggests no ubiquitous mechanistic link between leaf succulence and CAM *per se*, but rather that the co-occurrence of CAM and succulence should be confined only to leaves that display all cell succulence (e.g. Silvera *et al.*, 2005; Herrera, 2009).

This does not imply that species that develop leaf succulence via a central achlorophyllous hydrenchyma are precluded from having CAM or being facultatively CAM. For example, *Delosperma tradescantioides* can rapidly switch between C_3_ and CAM (Herppich *et al.*, 1996). This has been demonstrated in other storage succulents via intermediate isotopic values (e.g. Mooney *et al.*, 1977; Rundel *et al.*, 1999) and by more direct measures of CAM activity (Herppich *et al.*, 1996; Veste *et al.*, 2001). CAM-idling has been shown to be important for enduring drought and, subsequent to stomatal closure, the ability to capture and release respiratory CO_2_ allows continued light reaction activity that protects the photosynthetic apparatus from photoinhibition (Ting, 1985; Adams *et al.*, 1987; Griffiths *et al.*, 1989; Herrera, 2009). Some succulent species increase photosynthetic efficiency by CAM-cycling that can save up to 10% of daily assimilated CO_2_ that would otherwise be lost to the atmosphere (Patel and Ting, 1987; Herrera, 2009). These forms of CAM may capture only a small proportion of assimilated carbon through PEPc, and, when this is less than a third of total CO_2_ fixation, do not have δ^13^C values distinct from C_3_ plants (Winter and Holtum, 2002; Herrera, 2009), and hence cannot not be identified in isotopic surveys for CAM photosynthesis.

A range of Aizoaceae species were used to determine the role of increasing leaf succulence in prolonging gas exchange and maintaining photosynthetic integrity during leaf desiccation. The relationship between increasing succulence, CAM, and g_M_ was examined and the hypothesis was tested that the anatomy of Aizoaceae succulent leaves avoids low *g*_M_ and hence a strong dependence on CAM. This CAM–succulence relationship is contrasted between species from the Aizoaceae and Crassulaceae. The comparison was thus between plants that possess storage succulence and those that are all cell succulent.

## Materials and methods

### Species selection and propagation

For both the Aizoaceae and Crassulaceae, taxa were selected which showed a range of >5-fold differences in leaf succulence. Species from the Aizoaceae were selected from three of the four subfamilies in order to cover the phylogenetic and leaf morphological diversity of the group. Two species were selected from the Aizooideae with small, planar succulent leaves where the photosynthetic tissue is distributed throughout the leaf, termed ‘all cell succulence’ (*Galenia africana* L. and *Tetragonia fruticosa* L.); one species from the Mesembryanthemoideae (*Mesembryanthemum cordifolium* L.f.) with planar succulent leaves and ‘all cell succulence’; nine species from the Ruschieae (Ruschioideae), with subcylindrical to trigonous shaped succulent, rarely planar leaves, with an inner cortex of water-storing cells that are achlorophyllous, termed ‘storage succulence’ [*Antimima dasyphylla* (Schltr.) H.E.K.Hartmann, *Carpobrotus edulis* (L.) L. Bolus, *Delosperma echinatum* Schwantes, *D. tradescantioides* L. Bolus, *Drosanthemum speciosum* Schwantes, *Glottiphyllum depressum* N.E.Br., *G. longum* N.E.Br., *Lampranthus aureus* N.E.Br., and *Oscularia cedarbergensis* (L. Bolus), H.E.K.Hartmann]. Similarly, from the Crassulaceae, genetic and leaf morphological diversity within the group were selected, with most species selected from *Crassula*, since it is the largest genus of Crassulaceae in South Africa [*Adromischus fallax* Toelken, *Crassula atropurpurea* (Haw.) Dietr., *C. fascicularis* Lam., *C. ovata* (Mill.) Druce, *C. arborescens* (Mill.) Willd., *C. orbicularis* L., *C. pellucida* L., *C. sarmentosa* Harv., *C. multicava* Lem., *C. cordata* Thunb., *C. sarcocaulis* Eckl.&Zeyh., *C. dejecta* Jacq., *C. ericoides* Haw., *Sedum pachyphyllum* Rose, *Tylecodon singularis* (R.A.Dyer) Toelken, and *T. racemosus* (Harv.) Toelken]. The study included mostly field-collected plants from the winter-rainfall region of the Cape and a few from the summer-rainfall region of the Eastern Cape, South Africa. In addition, plants were procured from nurseries, which were established as a collection of potted plants at the University of Cape Town, Biological Sciences Department Greenhouse. Plants were grown in native or nursery soils, were well watered and were supplied with Long Ashton nutrient medium (Hewitt, 1966) modified to contain 2mm NaNO_3_ (pH 6.5) during pot establishment.

### Dehydration of excised shoots

A subsample of nine species from the family Aizoaceae (*A. dasyphylla*, *M. cordifolium*, *C. edulis*, *D. speciosum*, *G. africana*, *G. depressum*, *L. aureus*, *O. cedarbergensis*, and *T. fructicosa*) were used to determine the decline in photosynthetic rate (*A*), stomatal conductance (*g*_ST_), and potential photochemical efficiency (*F*_v_ */F*_m_) during a prolonged period of shoot dehydration. Shoots, or individual leaves in the case of large-leaved species, were excised from plants and the cut ends were sealed with nail varnish. Shoots or leaves were enclosed in a conifer chamber of a LI-6400 photosynthesis system (LI-COR Biosciences Inc., Lincoln, NE, USA). The chamber was illuminated from three sides using dichroic halogen spot lights (ECO-3000, Eurolux) such that all leaf surfaces received a phosynthetic photon flux density (PPFD) >1500 μmol m^–2^ s^–1^, leaf temperatures (estimated from energy balance using instrument software) controlled at 25±3 °C, and a vapour pressure deficit (VPD) <1.5 kPa. Gas exchange parameters were recorded at intervals during the next 10–20h. Measurements were made in triplicate and shoots or leaves were removed from the chamber between measurements, weighed, and kept under common (~25 °C, PPFD ~100 μmol m^–2^ s^–1^) conditions in the laboratory. The photosynthesis system was ‘matched’ before each data point was recorded and after *g*_ST_ and photosynthesis were stable at the prevailing conditions.

At the end of the experiment, leaf areas were measured by scanning cylindrical or ovoid leaves from two sides and triangular leaves from three sides. The accuracy of this method was checked by applying the same method to cylindrical, ovoid, and triangular shapes of known surface area. Gas exchange parameters were expressed on the basis of these multisided leaf areas (in contrast to the norm of expressing only on the area of one side of planar leaves).

Leaf or shoot turgid weights were recorded prior to the first measurement, immediately after the leaves or shoots were excised from well-watered plants. Dry weights were determined at the end of the experiments after drying plant material in an oven at 60 °C to constant weight. Leaf succulence was calculated as (turgid weight–dry weight)/dry weight, and leaf water content (WC) as (wet weight–dry weight)/turgid weight.

In a similar but independent experiment, the reduction photochemical efficiency of photosystem II (PSII; *F*_v_/*F*_m_) over a long period of dehydration was measured. Excised leaves or shoots were dark-adapted for 60min using leaf clips, and chlorophyll fluorescence parameters were recorded using a Walz PAM 2000 fluorometer (Walz, Effeltrich, Germany). After measurements, leaves of shoots were stored on a sunlit windowsill in the laboratory and measurements were repeated routinely for a period of 40 d. Measurements were made in duplicate for each plant species.

For each individual replicate, the decay in *A*, *g*_ST_, and WC over time (*t*) were fitted with the equation:


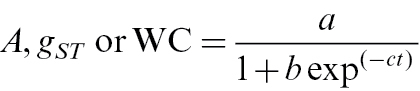
(1)

The declines in *A* and *g*_ST_ in response to decreasing leaf WC were fitted with the same equation, but WC was substituted for *t* in the equation above. The decrease in *F*_v_ */F*_m_ over time was fitted with the equation:


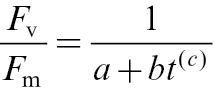
(2)

The parameters *a*, *b*, and *c* were fitted using least squared differences (using the Solver add-in of Microsoft Excel). Equation 1 was used to derive the time required to reduce *A* or *g*_ST_ by 50%, and WC by 10%. Equation 1 was also used to calculate the WC, and the reduction in turgid WC (turgid WC–WC), required to decrease *A* and *g*_ST_ by 50%. The latter measure accounts for the positive relationship between succulence and WC, and shows the effect of water loss from the initial turgid WC, and not from 100%. Equation 2 was used to calculate the time required to decrease *F*_v_ */F*_m_ by 10%. The choice of 10% or 50% for calculating the decline constants of *A*, *g*_ST_, WC, and *F*_v_ */F*_m_ was to ensure that estimates were not extrapolated beyond the measured data.

### *A*:*C*_i_ responses and mesophyll conductance determinations

Individual leaves of shoots were excised from the subsample of nine Aizoaceae species and cut ends were sealed into water-filled Eppendorf tubes using multiple wraps of Parafilm (Brand, Wertheim, Germany). These were then enclosed in a conifer chamber (Licor 6400-005) of a Licor 6400 photosynthesis system with chamber conditions set as before. The response of *A* to intercellular CO_2_ concentration (*C*_i_) was constructed according to Long and Bernacchi (2003) and individual replicate (*n* ≥3) curves fitted according to von Caemmerer (2000). Fitted parameters were used to generate *A*:*C*_i_ responses with points at 2 Pa intervals. *g*_M_ was then determined according to the constant *J* method (Warren, 2006), where the rate of linear electron transport (*J*_*a*_) was calculated from generated gas exchange data according to Equation 3.


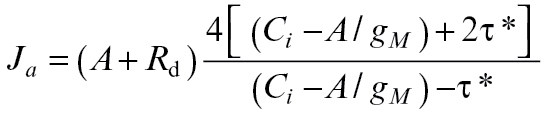
(3)

Where *A*=photosynthetic rate, *R*_d_=dark respiration, *C*_i_=intercellular CO_2_ partial pressure, *g*_M_=mesophyll conductance, and τ*=the photorespiratory compensation point. The value of *g*_M_ was obtained by iteratively changing the value between the limits of 0.01 μmol m^–2^ s^–1^ Pa^–1^ and 10 μmol m^–2^ s^–1^ Pa^–1^_._ The *g*_M_ value was selected that minimized the variance between *J*_a_ values determined at each *C*_i_ value (2 Pa intervals, between 90 Pa and 130 Pa) and the average *J*_a_ value calculated over this same range in partial pressures. This method of calculating *g*_M_ has acknowledged limitations (Pons *et al.*, 2009; Tholen *et al*., 2013), but given that the measurements were made on a range of species within a single family and that the plants were all grown under common conditions, the method retains its value for the interspecific comparison made in this study.

In some species it was possible to align the fibre optic of the fluorometer with leaves enclosed in a modified conifer chamber during the construction of *A*:*C*_i_ curves. This modification involved replacing the hinged ‘conifer chamber’ lid with a Perspex plate equipped with a port for the fibre optic probe. In these cases, simultaneous gas exchange and PSII fluorescence measurements were used to confirm that ФPSII was constant when *C*_i_ ranged from 90 Pa to 130 Pa.

### Leaf δ^13^C isotopic ratios and nitrogen concentration

Whole leaves were removed from well-watered plants for the entire Crassulaceae and Aizoaceae species collection (*n*=3) at ~10:00h. Leaf areas, and fresh and dry weights were recorded, and leaves were oven-dried at 60 °C to constant weight. Dried leaves were ground to a homogeneous powder using a Wiley mill with a 0.5mm mesh (Arthur H. Thomas, CA, USA) and weighed into 8×5mm tin capsules (Elemental Microanalysis Ltd, Devon, UK). Additional leaves from well-watered plants and plants dehydrated for 1 or 2 weeks were used to make extracts of leaf carbohydrates. Fresh leaves collected at ~10:00h were rapidly transferred into chilled 80% methanol, homogenized in a mixer-mill (Retsch MM 200, Retsch, Haan, Germany), and centrifuged in a microfuge. The supernatant was transferred to ion exchange columns for the separation of the neutral fraction (mostly carbohydrate) using a modification of the method of Atkins and Canvin (1971). The column was filled with a mixed bed ion-exchange resin (Dowex Marathon MR-3, Sigma-Aldrich, St. Louis, MO, USA) with deionized water as the mobile phase. The eluted neutral fraction was collected, air-dried under an N_2_ stream, and re-dissolved in 1ml of water. Aliquots (50 μl) of this sample were placed into mass spectrometer tin capsules and oven-dried at 65 °C. The neutral fraction of the methanol extraction contained no N, demonstrating that the carbohydrates had been effectively separated from amino compounds.

Additional leaves harvested from well-watered plants in the Aizoaceae were, where possible, separated into achlorophyllous hydrenchyma and chlorophyllous mesophyll tissue using a sterile scalpel blade. Separated tissues were oven-dried, weighed, milled, and subject to elemental analysis to determine tissue N concentrations. The stable carbon isotope ratios (δ^13^C) and tissue N concentrations for the foliar samples and the δ^13^C values for the carbohydrate samples were determined using a Thermo Flash EA 1112 series elemental analyser (Thermo Electron Corporation, Milan, Italy) and Delta Plus XP isotope ratio mass spectrometer (Thermo Electron Corporation).

### Estimates of photosynthetic and achlorophyllous cross-sectional areas

Fresh leaves taken from a subset of 10 species from the Crassulaceae and 11 species from the Aizoaceae were hand sectioned with a razor blade and photographed with a light microscope (Nikon, SMZ 1500). Digital micrographs were converted to 8-bit black and white images, and photosynthetic and achlorophyllous areas were measured using the ‘Analyse Particles’ function in Image-J (Version 1.44p).

## Results

In order to retain graphical clarity, the responses of *A*, *g*_ST_, WC, and *F*_v_ */F*_m_ to shoot excision and *A*:*C*_i_ responses are presented for select examples of species in the Aizoaceae. Data for the remaining species are given in the Supplementary Figures available at *JXB* online.

### Dehydration of excised shoots

**Fig. 1.**
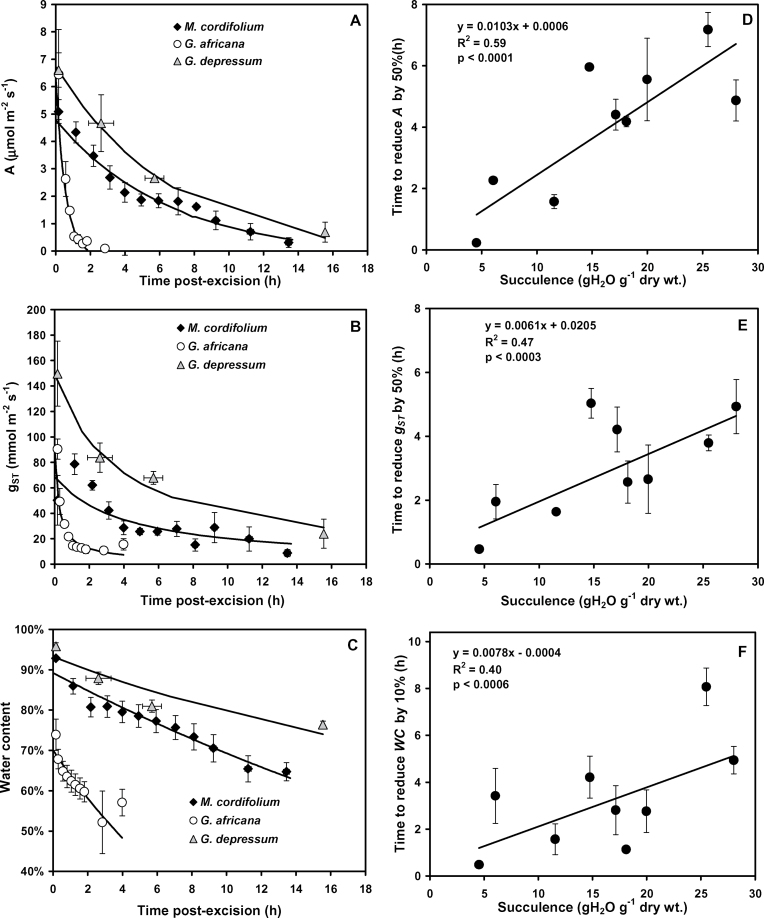
Decline over time in (A) average photosynthesis (*A*), (B) stomatal conductance (*g*_ST_), and (C) water content (WC) for selected examples of Aizoaceae species following leaf or shoot excision. Average time required to decrease (D) *A* and (E) *g*_ST_ by 50%, and (F) WC by 10%, for nine Aizoaceae species (*n*=3; mean ±SE). Linear regression lines with coefficients of determination and significance of correlations are shown. Time response data for *A. dasyphylla*, *C. edulis*, *D. speciosum*, *L. aureus*, *O. cedarbergensis*, and *T. fruticosa* are available in Supplementary Fig. S1 at *JXB* online.

*A*, *g*_ST_, and WC declined over time after leaf or shoot excision, were non-linear, and varied between species (Fig. 1A–C; Supplementary Fig. S1 at *JXB* online). The time required to reduce *A* and *g*_ST_ by 50% (*t*_50%_), and WC by 10% (*t*_10%_), was calculated from these curves and showed a linear response to increasing succulence (Fig. 1D–F). The most succulent species had *t*_50%_ values for *A* and *g*_ST_ that were >10-fold larger than those of the least succulent species. The *t*_50%_ values for *A* were higher than those for *g*_ST_, indicating that photosynthesis continued despite changes in *g*_ST_. This is due to a curvilinear relationship between *A* and *g*_ST_, particularly at high values of *g*_ST_ (data not shown). Water loss was sustained for longer from more succulent leaves, which also had the highest WC, and lost the initial 10% of leaf WC 10-fold more slowly than the least succulent species.

**Fig. 2.**
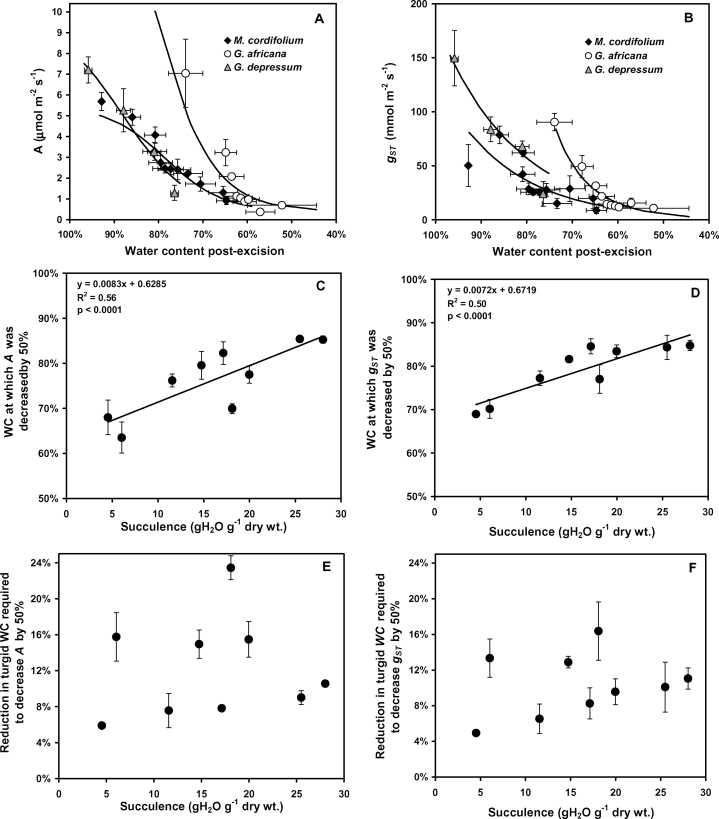
(A and B) Decline in average photosynthesis (*A*) and stomatal conductance (*g*_ST_) in response to water content (WC) for selected examples of Aizoaceae species following leaf or shoot excision. (C and D) Average WC at which *A* and *g*_ST_ were reduced by 50% for nine Aizoaceae species. (E and F) Average reduction in turgid WC required to decrease *A* and *g*_ST_ by 50% for nine Aizoaceae species (*n*=3; mean ±SE). Linear regression lines with coefficients of determination and significance of correlations are shown. Water content response data for *A. dasyphylla*, *C. edulis*, *D. speciosum*, *L. aureus*, *O. cedarbergensis*, and *T. fruticosa* are available in Supplementary Fig. S2 at *JXB* online.

The manner in which *A* and *g*_ST_ declined with WC was non-linear and varied between species (Fig. 2A, B; Supplementary Fig. S2 at *JXB* online). The WC at which *A* and *g*_ST_ were decreased by 50% was strongly correlated to leaf succulence (Fig. 2C, D) and showed that the most succulent species were the least tolerant of leaf dehydration in comparison with the least succulent species. There was no correlation between succulence and the decline in turgid leaf WC required to halve *A* (*P* > 0.5) or *g*_ST_ (*P* > 0.11; Fig. 2E, F). This expression accounts for the fact that the turgid WC of leaves varied with the degree of succulence. This shows that both *A* and *g*_ST_ were decreased by 50% when turgid *WC* was decreased by between 6% and 16% (average of 11%) across all the Aizoaceae species sampled. Only *L. aureus* tolerated more severe dehydration and required a reduction in WC of 23% to halve photosynthesis (Supplementary Fig. S2).

**Fig. 3.**
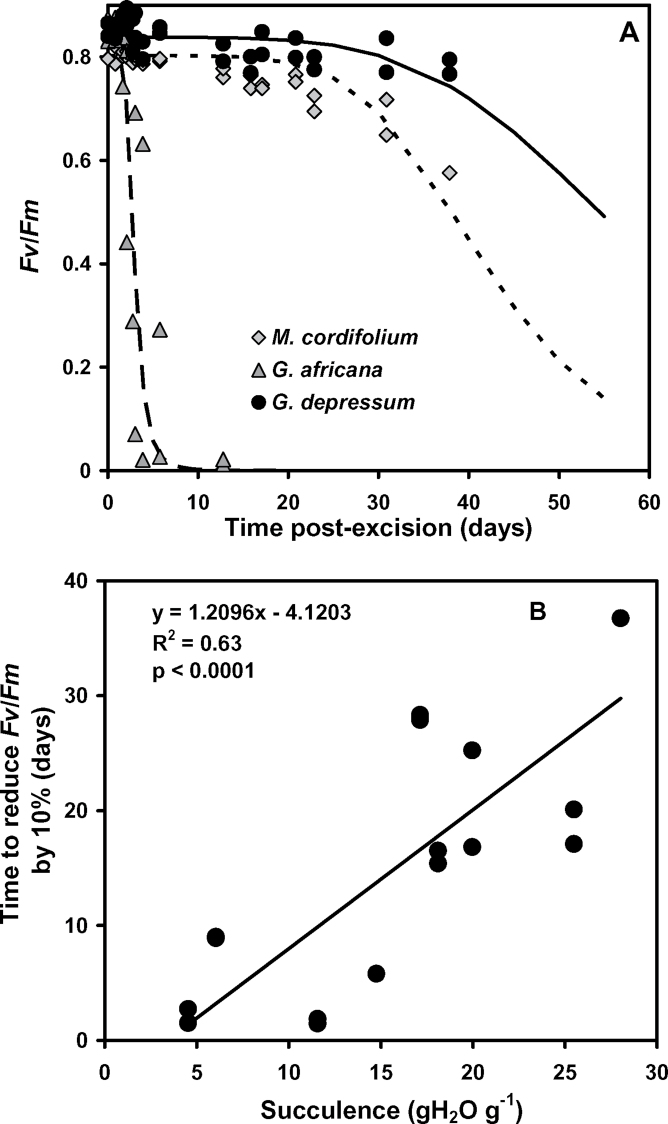
(A) Decline in efficiency of PSII (*F*_v_ */F*_m_) for individual replicates of selected examples of Aizoaceae species following leaf or shoot excision. (B) Time required to decrease *F*_v_ */F*_m_ by 10% for nine Aizoaceae species (*n*=2). For each individual, replicate data are shown. Linear regression lines with coefficients of determination and significance of correlations are shown. *F*_v_ */F*_m_ response data for *A. dasyphylla*, *C. edulis*, *D. speciosum*, *L. aureus*, *O. cedarbergensis*, and *T. fruticosa* are available in Supplementary Fig. S3 at *JXB* online.

Similarly, the decline in potential PSII photochemical efficiency (*F*_v_ */F*_m_) differed between species (Fig. 3A; Supplementary Fig. S3 at *JXB* online). However, a much longer dehydration period was required to affect *F*_v_/*F*_m_ than either *A* or *g*_ST_, and the most succulent species exhibited very little change in *F*_v_ */F*_m_ over a period of up to 20 d. As for *A* and *g*_ST_, however, the derived times required to reduce *F*_v_ */F*_m_ by 10% (*t*_10%_) were also positively correlated with increasing succulence, such that the most succulent species had a *t*_10%_ of 37 d, nearly 18-fold longer than the least succulent species (Fig. 3B).

### Leaf δ^13^C isotopic ratios

**Fig. 4.**
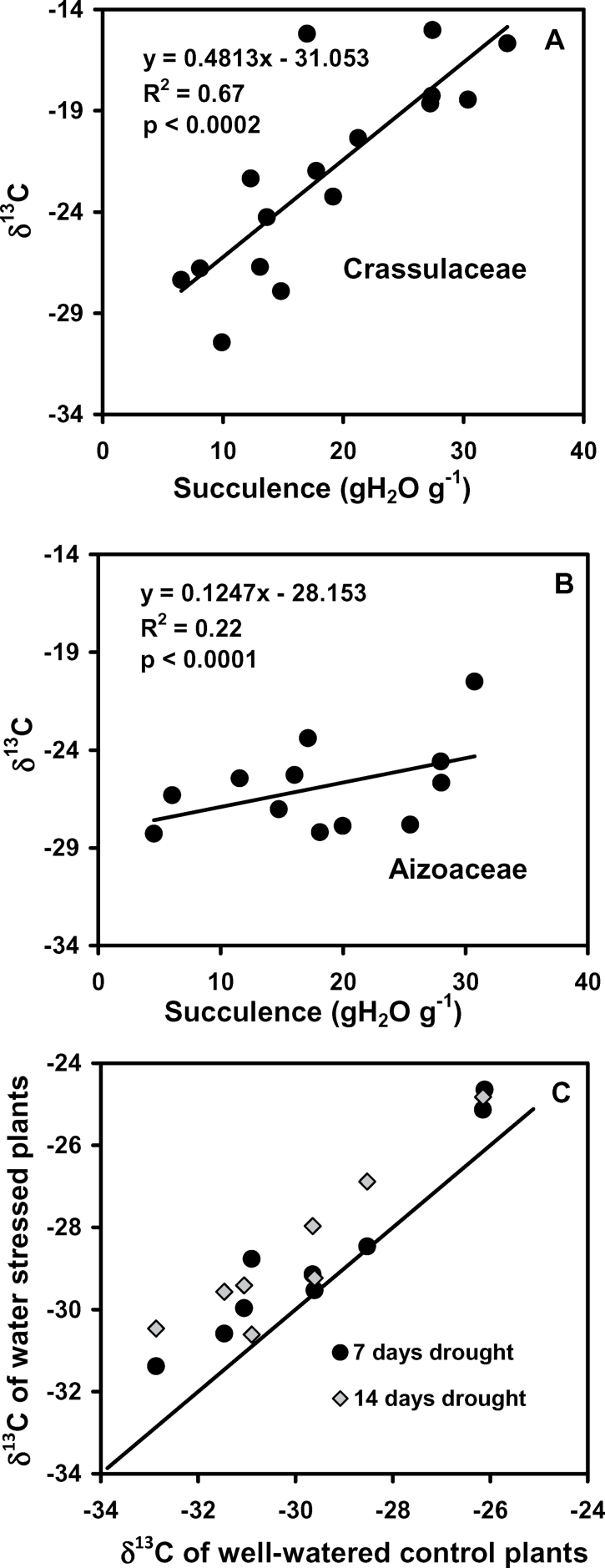
δ^13^C (‰) values for well-watered leaves for species from (A) Crassulaceae (*n*=16) and (B) Aizoaceae (*n*=12). Linear regression lines with coefficients of determination and significance of correlations are shown. (C) Response of δ^13^C values for select species from the Aizoaceae that were well watered or subject to water stress for 7 d or 14 d.

The δ^13^C values for leaves harvested from the 16 well-watered Crassulaceae species were positively correlated to leaf succulence (Fig. 4A). The range of values spanned from those that would be typical of C_3_ to CAM plants (Winter and Holtum, 2002). A similar relationship constructed for 12 of the Aizoaceae species showed a 4-fold weaker relationship (i.e. slope), and all values fell within the range typical of C_3_ plants or where CAM contributes only a small proportion to total carbon assimilation (Fig. 4B). To test if CAM could be induced by water stress, the δ^13^C values of carbohydrates extracted from well-watered leaves were compared with those from water-stressed plants. Water stress made δ^13^C less negative, by on average 1.0‰ and 1.5‰ after 7 d and 14 d, respectively (Fig. 4C). This small increase in δ^13^C is more likely to reflect the effect of decreased *g*_ST_ on gas exchange than a switch to CAM, which should have a much larger effect on the δ^13^C of recently assimilated carbohydrates.

### Photosynthetic cross-sectional area and leaf N content

**Fig. 5.**
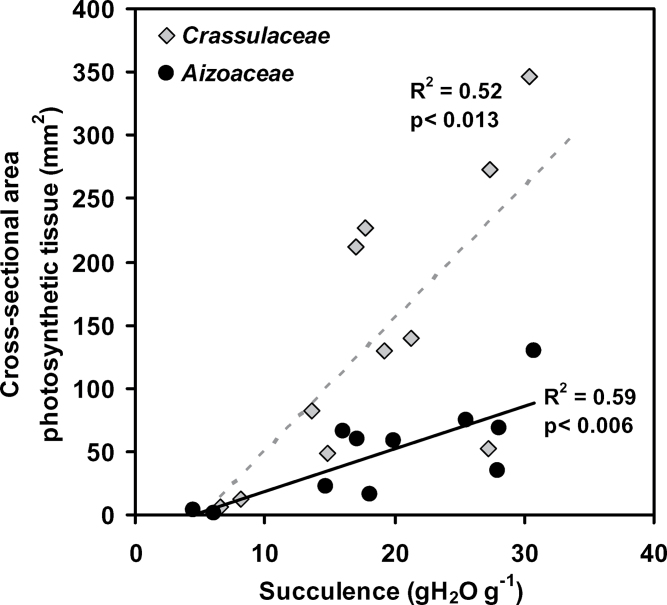
Variation in cross-sectional area of chlorophyllous tissue of Crassulaceae and the Aizoaceae species (*n*=11 species for both) with succulence. Linear regression lines with coefficients of determination and significance of correlations are shown.

Leaf N concentrations of neither the achlorophyllous hydrenchyma nor the photosynthetic mesophyll (Table 1) were correlated to succulence (all *R* ^2^ < 0.05, data not shown). The N contents of the achlorophyllous hydrenchyma were high, being on average (across species) 26±6% of the total leaf N content, suggesting that this could be an important N store in these leaves. The cross-sectional area of photosynthetic tissue was linearly correlated to succulence in both the taxonomic groups; however, the slope of the relationship was 3-fold higher in the Crassulaceae than in the Aizoaceae (Fig. 5). The difference in the slopes results from the fact that the more succulent species of the Aizoaceae possess achlorophyllous hydrenchyma while the less succulent Aizoaceae species and the Crassulaceae exhibit chlorophyllous ‘all cell succulence’.

**Table 1.** Leaf succulence and N concentrations (%, w/w) measured separately for achlorophyllous hydrenchyma and photosynthetic tissue (where possible) for nine species of Aizoaceae

Species	Succulence (g H_2_O g^–1^ dry weight)	Achlorophyllous hydrenchyma N (%)	Chlorophyllous mesophyll N (%)
*Antimima dasyphylla*	6.0±0.9	–	1.97±0.38
*Mesembryanthemum cordifolium*	17.1±1.6	–	1.78±0.12
*Carpobrotus edulis*	20.0±2.3	3.83±0.77	2.91±0.04
*Drosanthemum speciosum*	14.8±0.6	0.73±0.06	1.15±0.12
*Galenia Africana*	4.5±0.5	–	2.98±0.23
*Glottiphyllum depressum*	28.0±3.4	0.59±0.12	1.73±0.14
*Lampranthus aureus*	18.1±0.6	2.51±0.15	3.95±0.30
*Oscularia cedarbergensis*	25.5±3.8	1.79±0.48	2.45±0.09
*Tetragonia fruticosa*	11.6±2.5	–	1.23±0.06

Values are means ±SE (*n*=3).

### *A*:*C*_i_ responses and mesophyll conductance

**Fig. 6.**
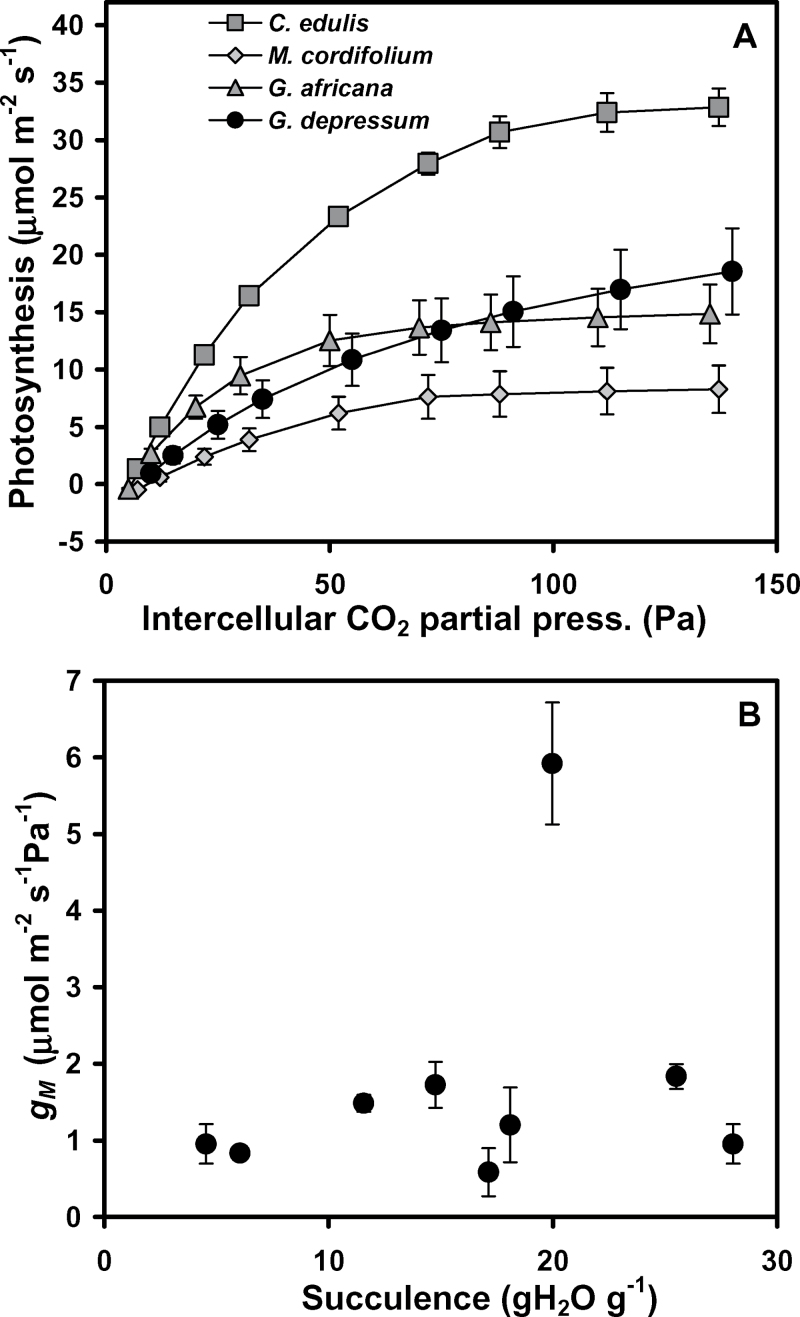
(A) *A*:*C*_i_ responses of selected examples of Aizoaceae species. Fitted equations for each replicate were used to interpolate data to a common series of *C*_i_ values for the calculation of means and standard errors. (B) Derived mesophyll conductance values (*g*_M_) for nine Aizoaceae species. For each species *n* ≥3, and vertical bars are standard errors. *A*:*C*_i_ responses for *A. dasyphylla*, *D. speciosum*, *L. aureus*, *O. cedarbergensis*, and *T. fruticosa* are available in Supplementary Fig. S6 at *JXB* online).

*A*:*C*_i_ responses for the Aizoaceae (Fig. 6A; Supplementary Fig. S4 at *JXB* online) produced a wide range of *V*_cmax_ (16–70 μmol m^–2^ s^–1^) and *J*_max_ values (41–148 μmol m^–2^ s^–1^; Supplementary Table S1 at *JXB* online) that were significantly different between the species (*V*_cmax_, *F*_8,22_=11.3, *P* < 0.0001; *J*_max_, *F*_8,22_=8.0, *P* < 0.0001). These parameters were not correlated to leaf succulence (all *R* ^2^ >0.1; data not shown). The *g*_M_ values derived from the CO_2_-saturated portions of the *A*:*C*_i_ responses were significantly different between species (*F*_8,22_=4.1, *P* < 0.004) and ranged from 0.59 μmol m^–2^ s^–1^ Pa^–1^ to 1.73 μmol m^–2^ s^–1^ Pa^–1^, excluding the apparent outlier value of 5.9 μmol m^–2^ s^–1^ Pa^–1^ for *C. edulis* (Fig. 6B). The *g*_M_ values were not correlated to leaf succulence (*P* > 0.11).

## Discussion

As hypothesized, increasing succulence in the Aizoaceae was not accompanied by a decrease in mesophyll conductance (*g*_M_). The range of *g*_M_ values of the Aizoaceae that were measured were generally higher than those reported for the CAM succulent *Kalanchoe daigremontiana* (~0.5 μmol m^–2^ s^–1^ Pa^–1^; Maxwell *et al.*, 1997; Griffiths *et al.*, 2007). The present data are, however, expressed on a total leaf area basis since several leaves were cylindrical or triangular in cross-section, rather than as usual for single surfaces of planar leaves, and thus equivalent values for *K. daigremontiana* would be ~0.25 μmol m^–2^ s^–1^ Pa^–1^. The present values mostly fall withinthe range of *g*_M_ values reported for C_3_ species (with correction to consider both leaf surfaces: ~0.38–2.18 μmol m^–2^ s^–1^ Pa^–1^; Flexas *et al.*, 2007). This high *g*_M_ in the Aizoaceae is remarkable considering how succulent some of these leaves are, and that these succulent leaves largely lack mesophyll airspaces, which contribute to high *g*_M_ in many C_3_ species. The relatively high *g*_M_ of the Aizoaceae is, however, readily explained by the fact that anatomically the photosynthetic tissue is in a thin band adjacent to the leaf surface. Avoiding the limitations of low *g*_M_ via anatomical modifications is not unique to the Aizoaceae, and stomatal crypts play a similar role in *Banksia* leaves with high dry mass per area (Hassiotou *et al.*, 2010). Additionally, limiting the water-demanding photosynthetic tissue to a thin layer and associating this with three-dimensional venation (Ogburn and Edwards, 2013) would maintain favourable hydraulic path lengths, whilst allowing the development of succulent leaves. This hydraulic supply would be important for the uptake and recharge of photosynthetic water storage tissue and to service photosynthetic *g*_M_ and the water loss from photosynthetic surfaces (Griffiths, 2013).

The Aizoaceae lack a strong dependence on CAM, as was evident from the δ^13^C isotope values, which were all more negative than –21‰, which is the cut-off value determined for CAM species (Winter and Holtum, 2002). The average δ^13^C value was –29.6‰, which indicates that >60% of carbon gain would have occurred diurnally via the C_3_ cycle (Winter and Holtum, 2002). The fact that the δ^13^C values only increased by 1.5‰ after 14 d of water stress indicates that the high δ^13^C values relative to those of typical CAM plants were not due to the well-watered conditions under which the plants were generally maintained, and that CAM is not induced in these species by water stress. This small shift in δ^13^C in response to water stress could be accounted for by changes in the *C*_i_/*C*_a_ induced by stomatal closure, as in C_3_ species (Farquhar *et al.*, 1989).

The succulent Aizoaceae leaves utilized the water stored in the achlorophyllous hydrenchyma to prolong positive gas exchange and allowed leaves to withstand long periods of drought. This photosynthetic buffering and the ability to endure drought have been demonstrated in numerous succulent species and not just those with achlorophyllous hydrenchyma (e.g. Nobel, 1976; Hoffman *et al.*, 2009). Interestingly, in the Aizoaceae, the more succulent species were least physiologically tolerant of lowered leaf tissue water content. The positive correlation between succulence and the WC required to halve *A* and *g*_ST_ meant that the gas exchange of the most succulent species responded at the highest water contents. When the differences in initial turgid WC were accounted for, it was apparent that when the initial WC was decreased by 6–16%, the photosynthetic and stomatal response were evident irrespective of the degree of leaf succulence. Hence, the mechanism for sustained gas exchange was not due to tolerance of dehydration, but the most succulent species had a large volume of water to buffer dehydration. Maintenance of photosynthetic tissue water relations at the expense of water loss from the hydrenchyma has been demonstrated for various species (Schmidt and Kaiser, 1987; Herrera *et al.*, 2000; Nobel, 2006), and interspecific differences in this might account for the range in WC loss required to produce the observed gas exchange responses.

Once positive gas exchange had ceased, the capacity for photosynthesis was maintained for long periods of drought and was directly correlated to the degree of leaf succulence.

Most succulent species reached *t*_50%_ for *A* within 7h, but *t*_10%_ for *F*_v_/*F*_m_ only occurred after 38 d, indicating that the energy provision from the photosynthetic lights reactions continued even in the absence of net CO_2_ acquisition. Maintenance of photochemistry is likely to be important for energy provision to offset maintenance costs. Furthermore, a functioning photochemical system might enable rapid resumption of carbon assimilation after rainfall events. Such rapid gas exchange responses to water inputs have been demonstrated in various leaf succulents including *Ruschia caroli* (Aizoaceae; Midgley and van der Heyden, 1999), *Mesembryanthemum pellitum* (Aizoaceae), and *Othonna optima* (Asteraceae; Eller *et al.*, 1993). The maintenance of photochemical integrity may be aided by CAM-idling, or drought-induced low-level CAM, which has been demonstrated in *D. tradescantioides* (Aizoaceae; Herppich *et al.*, 1996). CAM-idling does not contribute to overall carbon assimilation and hence does not result in discernible CAM δ^13^C signal (Winter and Holtum, 2002). Consequently, the importance of these mechanisms may have been overlooked in many succulent species that have C_3_-like isotopic values and would not have been discernible with the present measurements.

The species distribution of the Aizoaceae in the GCFR overlaps that of the Crassulaceae (von Willert *et al.*, 1990; Jürgens, 1997; Goldblatt and Manning, 2002), many of which are constitutive CAM plants (e.g. Mooney, 1977). Of the ~659 species of the Aizoaceae, ~524 (80%) are endemic to the winter rainfall GCFR. In contrast, only 35 (26%) of 134 species of Crassulaceae are endemic to this region (Goldblatt and Manning, 2002). Where the Crassulaceae are found in the GCFR, their centre of diversity is in a region of higher mean annual precipitation (200–290mm) and less severe summer drought in the Cedarberg mountains and surrounding valleys and the Little Karoo (Jürgens, 1997; Mucina *et al.*, 2006; Rebelo *et al.*, 2006). The centre of diversity for the succulent Aizoaceae (subfamilies Ruschioideae and Mesembranthemoideae) occurs at lower (90–164mm) strongly winter rainfall, in the Richtersveld and Knersvlakte (Hartmann, 1991). It is speculated that the peripheral photosynthetic tissue and achlorophyllous hydrenchyma of the succulent Aizoaceae may be especially suited to the wet winters and dry summers found in this region. The greater dependence on CAM of the Crassulaceae may be associated with the fact that these plants receive moisture and grow during the hot summers.

The onset of the growing season in the Succulent Karoo is early May, reaching the mid-growing season in August, with growth ceasing by early January (Wessels *et al.*, 2011). The combination of cold and rain eliminates water stress for the winter and some of the spring period, during which time CAM would not be beneficial. Under this climate scenario, the role of succulence in the Aizoaceae may be to extend the growing season into the drier and warmer period of early summer, after which the plants may become dormant during the hot dry period of late summer. Thus, the engagement of C_3_ photosynthesis interspersed with periods of dormancy in net CO_2_ acquisition, with or without weak CAM or CAM-idling, is an alternative to constitutive CAM in these seasonally arid environments. A similar habitat differentiation based on rainfall has been observed for CAM plants in Madagascar. Here, CAM plants occur in dry climatic zones or in dry micro-habitats, whereas facultative CAM physiotypes occur in less stressful environments (Kluge *et al.*, 2001). Because CAM is a more energetically demanding photosynthetic system than C_3_ (von Willert *et al.*, 1992; Winter and Smith, 1996; Lüttge 2004), it might not be ubiquitously suitable for Mediterranean climates. CAM is particularly beneficial in hot conditions where it enables positive C acquisition, unlike C_3_ photosynthesis which becomes increasingly compromised by photorespiratory CO_2_ losses at higher temperatures (Nobel, 1991). Thus, although both the Aizoaceae and Crassulaceae tolerate drought, the seasonality of rainfall and the growing seasons may result in different photosynthetic strategies being beneficial.

While succulence is commonly associated with retaining tissue hydration, an additional limitation of seasonally arid environments is the availability of soil nutrients, which require water to become soluble and accessible to plants (Cui and Caldwell, 1997). This limitation is particularly pertinent in shallow soils or where roots are confined to rock crevices and small soil pools, as is the case for many succulents in the CFR (Midgley and van der Heyden, 1999). The extension of the period over which gas exchange can occur during water stress can only benefit growth if there is a simultaneous availability of nutrients for new tissue expansion. It is likely that succulent plants increase nutrient uptake during periods of water availability and store those nutrients. This storage of nutrients is an additional role for the achlorophyllous hydrenchyma in the succulent Aizoaceae and explains the high N contents measured in these tissues.

## Supplementary data

Supplementary data are available at *JXB* online.

Figure S1. Decline over time in average photosynthesis (*A*), stomatal conductance (*g*_ST_), and water content (WC) for the indicated Aizoaceae species following leaf or shoot excision (*n*=3; mean ±SE).

Figure S2. Decline in average photosynthesis (*A*) and stomatal conductance (*g*_ST_) in response to water content (WC) for the indicated Aizoaceae species following leaf or shoot excision (*n*=3; mean ±SE).

Figure S3. Decline in initial *F*_v_/*F*_m_ over time for the indicated Aizoaceae species following leaf or shoot excision. For each species *n*=2 and hence individual replicate and not average data are presented.

Figure S4. *A*:*C*_i_ responses for the indicated Aizoaceae species. For each species *n* ≥3, and vertical bars are standard errors.

Supplementary Data

## Abbreviations:

A: net photosynthetic rate
CAM: Crassulacean acid metabolism
*C*_i_: intercellular CO_2_ concentration
δ^13^C: stable carbon isotope ratio
*F*_*v*_/*F*_m_: potential PSII photochemical efficiency
*g*_ST_: stomatal conductance
*g*_M_: mesophyll conductance
*J*_a_: rate of linear electron transport
*J*_max_: maximum of rate of electron transport
PPFD: photosynthetic photon flux density
*R*_d_: dark respiration
τ*: photorespiratory compensation point
*V*_cmax_: Rubisco carboxylation
VPD: vapour pressure deficit
WC: leaf water content.
