# Supplementary material for: How succulent leaves of Aizoaceae avoid mesophyll conductance limitations of photosynthesis and survive drought
Source: J Exp Bot. 2013 Oct 14;64(18):5485–96. doi: 10.1093/jxb/ert314 (PMC3871808; doi:10.1093/jxb/ert314)
Supplement: Supplementary Data [file supp_64_18_5485__index.html]

How succulent leaves of Aizoaceae avoid mesophyll conductance limitations of photosynthesis and survive drought — How succulent leaves of Aizoaceae avoid mesophyll conductance limitations of photosynthesis and survive drought — Supplementary Data 

# How succulent leaves of Aizoaceae avoid mesophyll conductance limitations of photosynthesis and survive drought

## Supplementary Data

Data files

**Files in this Data Supplement:**

- Supplementary Data - Supplementary Data
