# Supplementary material for: How succulent leaves of Aizoaceae avoid mesophyll conductance limitations of photosynthesis and survive drought
Source: J Exp Bot. 2013 Oct 14;64(18):5485–96. doi: 10.1093/jxb/ert314 (PMC3871808; doi:10.1093/jxb/ert314)
Supplement: Supplementary Data [file supp_ert314_jexbot102707_file001.pdf]

How succulent leaves of Aizoaceae avoid mesophyll conductance limitations of photosynthesis and survive drought. *Brad S Ripley, Trevor Abraham, Cornelia Klak and Michael D Cramer*

SUPPLEMENTARY DATA

Table S1 Maximum of electron transport rate (J_{max}) and Rubisco carboxylation (V_{cmax}) values for nine Aizoaceae species. Values are means ± SE (n≥ 3).

**Species**	J_{max} (μmol m^{-2} s^{-1})	V_{cmax} (μmol m^{-2} s^{-1})
Antimima dasyphylla	51 ± 2	16 ± 2
Mesembryanthemum cordifolium	41 ± 10	20 ± 5
Carpobrotus edulis	158 ± 10	68 ± 3
Drosanthemum speciosum	69 ± 7	35 ± 3
Galenia africana	70 ± 17	60 ± 9
Glottiphyllum depressum	80 ± 21	24 ± 5
Lampranthus aureus	84 ± 13	57 ± 8
Oscularia cedarbergensis	79 ± 3	36 ± 2
Tetragonia fruticosa	68 ± 2	41 ± 4

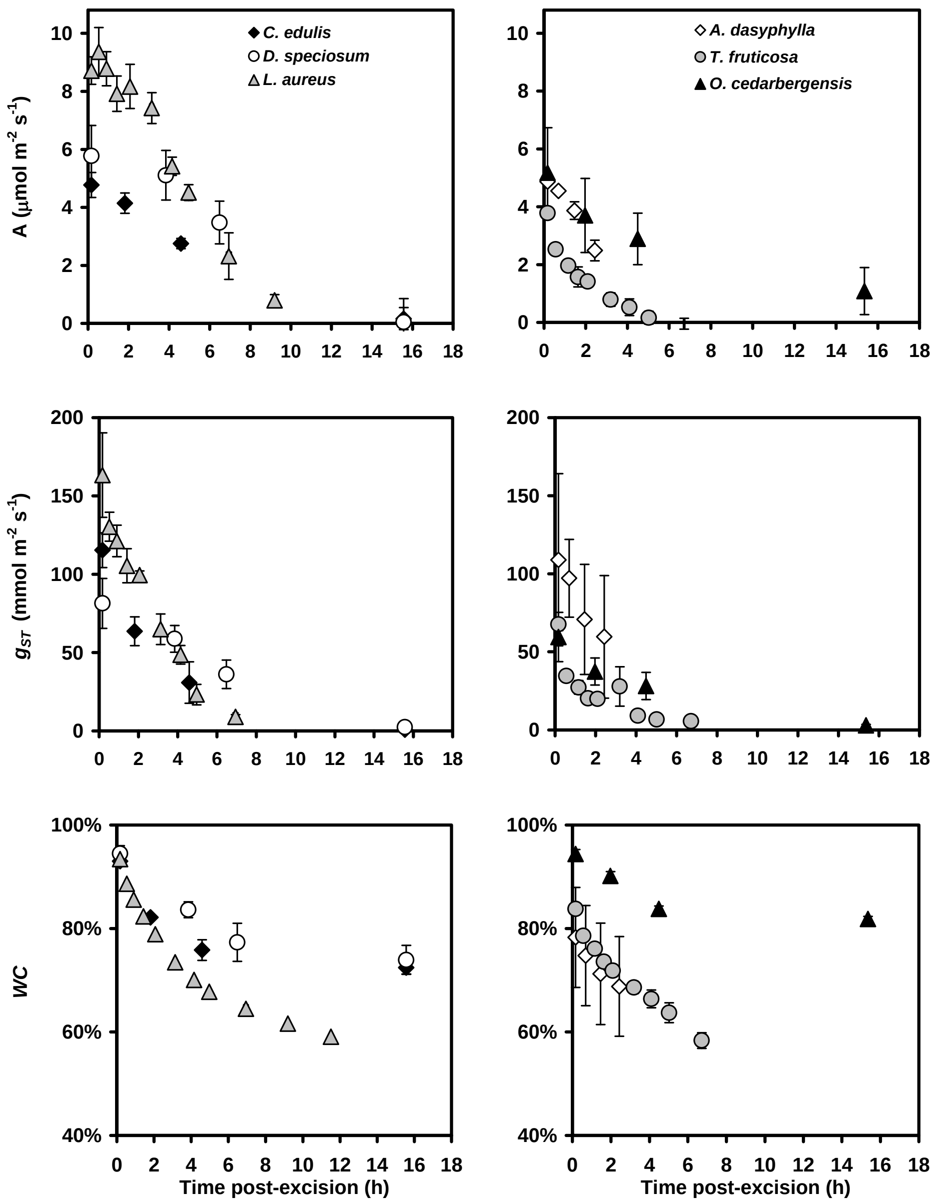

Figure S1 - Decline over time in average photosynthesis (*A*), stomatal conductance (g_{ST}) and water content (*WC*) for indicated Aizoaceae species following leaf or shoot excision (n = 3; mean ± SE).

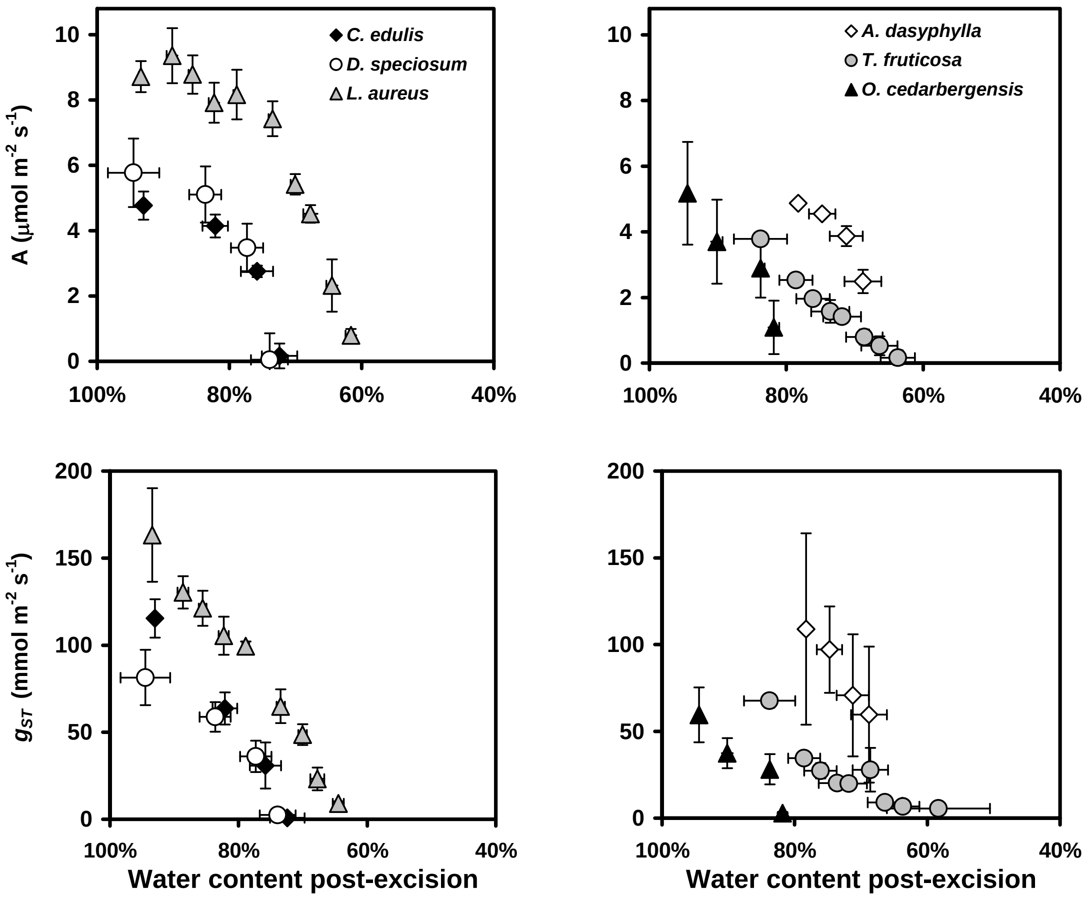

Figure S2 - Decline in average photosynthesis (*A*) and stomatal conductance (g_{ST}) in response to water content (WC) for indicated Aizoaceae species following leaf or shoot excision (n = 3; mean ± SE).

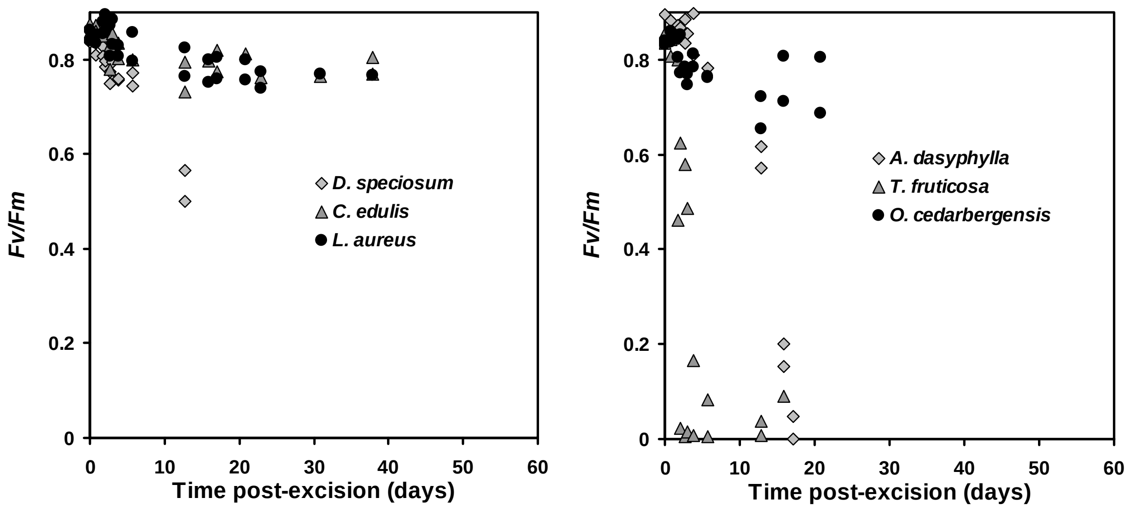

Figure S3 - Decline in initial Fv/Fm over time for indicated Aizoaceae species following leaf or shoot excision. For each species n= 2 and hence individual replicate and not average data are presented.

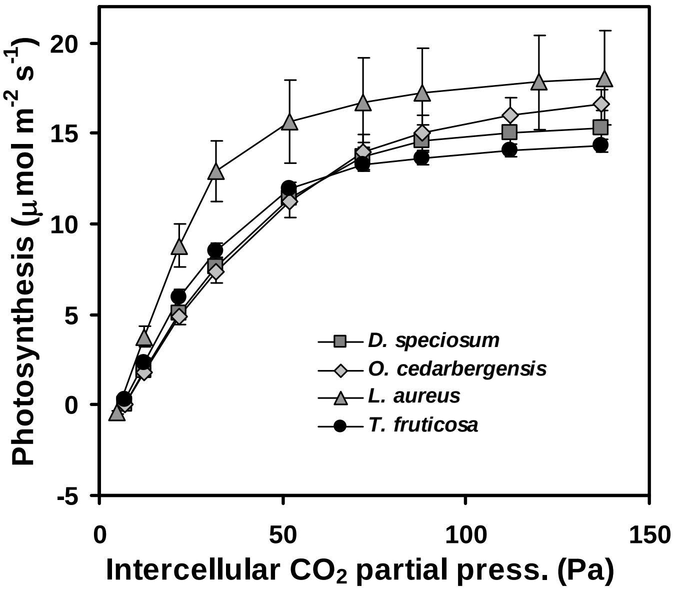

Figure S4 - *A:C_i* responses of for indicated Aizoaceae species. For each species n≥3 and vertical bars are standard errors. Fitted equations for each replicate were used to interpolate data to a common series of C_i values for the calculation of means and standard errors.
